# A modified mycobacterial growth inhibition assay for the functional assessment of vaccine-mediated immunity

**DOI:** 10.1038/s41541-024-00906-z

**Published:** 2024-07-02

**Authors:** Emil Joseph Vergara, Andy Cano Tran, Matthew J. Paul, Thomas Harrison, Andrea Cooper, Rajko Reljic

**Affiliations:** 1 Institute for Infection and Immunity, St. George’s University of London, London, UK; 2 Department of Infection, Immunity and Inflammation, University of Leicester, Leicester, UK

**Keywords:** Vaccines, Bacteriology, Live attenuated vaccines

## Abstract

The Mycobacterial growth inhibition assay (MGIA) is an ex-vivo assay used to measure the overall functional immune response elicited by infection or vaccination. In tuberculosis (TB) vaccine development, MGIA is a potentially important tool for preclinical evaluation of early-stage vaccine candidates to complement existing assays, and to potentially reduce the need for lengthy and costly pathogenic *Mycobacterium tuberculosis* (Mtb) animal challenge experiments. The conventional method of MGIA in mice entails directly infecting mixed cell cultures, most commonly splenocytes, from immunised mice with mycobacteria. However, this direct infection of mixed cell populations may yield unreliable results and lacks sufficient sensitivity to discriminate well between different vaccines due to the low number of mycobacteria-permissive cells. Here, we modified the assay by inclusion of mycobacteria-infected congenic murine macrophage cell lines as the target cells, and by measuring the total number of killed cells rather than the relative reduction between different groups. Thus, using splenocytes from *Mycobacterium bovis* BCG immunised mice, and J774 and MH-S (BALB/c background) or BL/6-M (C57Bl/6 background) macrophage cell lines, we demonstrated that the modified assay resulted in at least 26-fold greater mycobacterial killing per set quantity of splenocytes as compared to the conventional method. This increased sensitivity of measuring mycobacterial killing was confirmed using both the standard culture forming unit (CFU) assay and luminescence readings of luciferase-tagged virulent and avirulent mycobacteria. We propose that the modified MGIA can be used as a highly calibrated tool for quantitating the killing capacity of immune cells in preclinical evaluation of vaccine candidates for TB.

## Introduction

Assays that can demonstrate functional immunity are useful tools for preclinical vaccine research to demonstrate both the potential protective efficacy of a vaccine, and the underlying mechanism by which the generated immune response works. Currently, preclinical evaluation of vaccine candidates for tuberculosis (TB) utilises avirulent and virulent strains of mycobacteria in several animal models^1^. In the earliest phase of vaccine development, novel antigens, adjuvants, delivery systems, or a combination of all three are assessed for immunogenicity and protective efficacy in mice^1^. This approach can be very complex and costly especially since work with pathogenic *Mycobacterium tuberculosis* (Mtb) requires containment level 3 facilities (CL3), which may not be accessible to many laboratories. Accounting for statistical power, assessing the immunogenicity and protective efficacy of a vaccine candidate ordinarily requires the use of many mice.

There has been a movement towards using alternative methods for proof-of-concept testing of several biologicals in an effort to reduce animal use for preclinical R&D^2^. For TB vaccine research, this has led to efforts at developing assays to measure the capacity of immune cells from infected or vaccinated mice to restrict mycobacterial growth in vitro. One assay, referred to as mycobacterial growth inhibition assay (MGIA), has been in development for many years and, so far, demonstrated utility for assessing functional immune responses in mice, non-human primates (NHPs), cattle and humans^3–9^.

The conventional method of MGIA^10^ (convMGIA) is based on the infection of a mixed cell population obtained from samples such as whole blood^4,7^, PBMCs^3,4,8–10^, spleen^5,6,11,12^ or lungs^5^ with avirulent and virulent strains of mycobacteria. Viable mycobacteria are then quantified in the sample after a certain period using different bacteriologic assays. From an immunological perspective, mycobacteria-infected cells, most of which are macrophages, increase expression of major histocompatibility molecules (MHC) on their surface which allows recognition and targeting by antigen-specific conventional T cells. Despite its use by some groups and many attempts at optimising and standardising the assay^6,10^, MGIA still remains to be adopted for widespread use owing to issues with both its reproducibility^4^ and sensitivity.

Coculture assays have also been utilised to demonstrate the capacity of cellular immune responses to restrict the growth of mycobacteria in vitro^13–16^. Coculture experiments are widely used for the assessment of functional cell-mediated immunity against cancer cells^17–20^ and infectious agents such as viruses^21,22^ and intracellular bacteria^13–16,23,24^. These assays are based on the interaction between target cells expressing peptide-MHC complexes (pMHC) on the cell surface, and immune effector cells that can recognise displayed pMHCs on the surface of target cells. In infectious diseases research, target cells are infected with a virus or bacteria followed by coculture of splenocytes or purified immune effector cells such as conventional and unconventional T cells^14–16,21,22,24^. MHC molecules are subsequently recognised by cognate CD4 and/or CD8 T cells leading to T cell-mediated killing of target cells through cytokine- or cytotoxic-mediated T cell effector functions^25^.

Previous coculture experiments have utilised primary macrophages isolated from either the peritoneum (peritoneal macrophages, PMs) or bone marrow (bone marrow-derived macrophages, BMDMs) for use in coculture experiments with splenocytes or enriched T cell populations to demonstrate mycobacterial killing in vitro^13–16^. This approach, however, requires the use of additional animals and can be technically demanding especially for protocols that require differentiation of bone marrow progenitor cells to BMDMs. These protocols can also be time consuming since differentiation protocols of bone marrow progenitor cells to BMDMs take at least a week^13^, while isolation of PMs via thioglycolate injection through the peritoneum requires at least 3 to 5 days^15,16^. In addition, some published protocols used enriched populations of conventional CD4 or CD8 T cells to demonstrate mycobacterial restriction which adds another layer of complexity to the design and execution of these experiments due to the need for magnetic or FACS-based enrichment of CD4 or CD8 T cells.

Here, we developed a simplified coculture assay, herein referred to as modified MGIA (modMGIA), wherein macrophage cell lines from BALB/c (MH-S and J774) and C57Bl/6 (C57Bl/6-M) mice, both commonly used mouse strains for TB vaccine research, were used to enrich for mycobacteria-infected cells that can be targeted by effector cells (antigen-specific conventional T cells) obtained from the spleen. We posit that cell lines are convenient target cells due to their ease of use, availability, and abundance. More importantly, the use of cell lines eliminates the need for live animals to harvest primary cells. However, one key issue with cell lines is that they may behave differently from primary cells. As such, we initially characterised the permissiveness and utility of target cell lines in the context of mycobacterial infection. Afterwards, infected cell lines were cocultured with splenocytes from immunised and non-immunised mice. We demonstrated that BCG-mediated immunity can lead to restriction of mycobacterial growth in vitro to a greater effect than the conventional MGIA. We, therefore, propose that the modMGIA can be used as a tool for the preclinical evaluation of vaccine candidates for TB. The assay can also be explored for its utility in other intracellular pathogens such as bacteria and viruses.

## Results

### Low frequency of mycobacterial infection in spleens of healthy mice

**Fig. 1:**
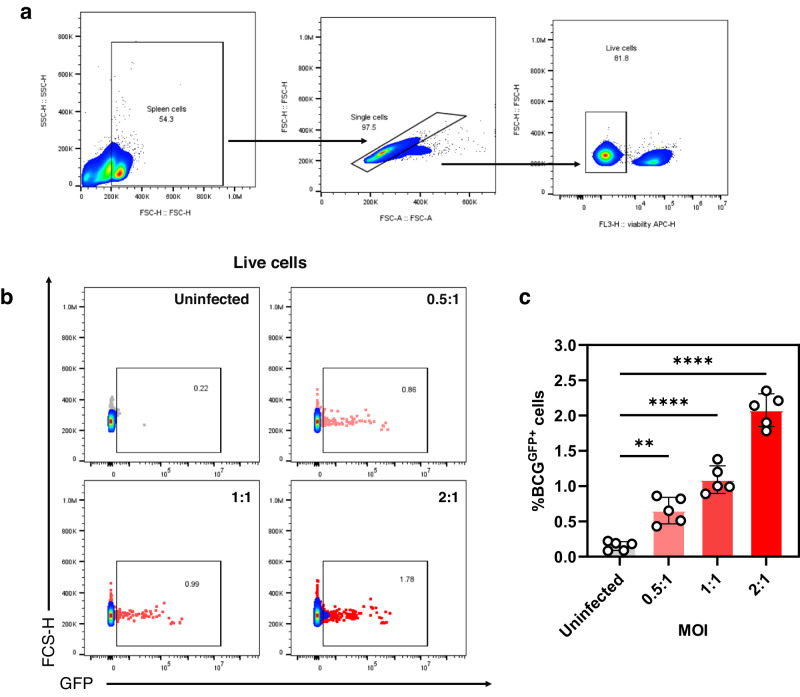
BCG infection of mouse splenocytes results in low frequency of infected cells. Splenocytes obtained from BCG naïve mice were infected with different MOI (0.5:1, 1:1, 2:1) of BCG^GFP^ for 24 h followed by flow cytometry to probe for BCG^GFP^ live cells. **a** Gating strategy, **b** representative dot plots and **c** bar graph showing % BCG^GFP+^ live cells. Error bars indicate SEM. Statistical significance was calculated using one-way ANOVA followed by Tukey’s multiple comparison test (*P < 0.05, **P < 0.01, ***P < 0.001).

Most published protocols for conventional MGIA in mice used splenocytes obtained from unimmunised and immunised mice where splenocytes were directly infected with non-pathogenic or pathogenic mycobacteria, followed by quantification of mycobacterial load in samples after an incubation for 96-120 hrs^5,6,11,12^. We hypothesised that the marginal differences seen in conventional MGIA assays could be due to the low level of infection by mycobacteria in a mixed cell population like the spleen. As the capacity of conventional T cells to restrict mycobacterial growth is an important consideration for preclinical evaluation of vaccines, having sufficient infected target cells (e.g., macrophages or dendritic cells) is crucial for the assay to demonstrate reliable results. To test this, spleens obtained from healthy mice were infected with different MOI (0.5:1, 1:1 and 2:1; bacteria:cells) of BCG^GFP^ followed by flow cytometry analysis 24 h post infection to determine the proportion of infected cells. Healthy mice were used to ensure that mycobacteria-infected cells are not subject to targeting by effector T cells, which can potentially confound findings. As seen in Fig. 1c, we observed that even for the highest MOI (2:1), only 2.076% (±0.190) of all cells in the spleen get infected with BCG. This was further reduced to 1.092% (±0.160) and 0.654% (±0.153) for the 1:1 and 0.5:1 MOI, respectively. Overall, we observed that splenocytes from healthy mice have a low infection threshold for mycobacteria.

### Permissiveness and utility of J774 and MH-S cell lines to mycobacterial infection

Since we based the premise of our modified assay on enriching for target cells for killing by the effector spleen cells, we initially screened two macrophage cell lines, MH-S and J774, for potential applicability for coculture assays. Unlike previous coculture assays which used primary cells obtained from mice, we chose to utilise congenic macrophage cell lines as target cells owing to the convenience, availability, and abundance of murine cell lines. Importantly, this eliminates the need to sacrifice mice for isolation of primary macrophages as well. As cell lines may behave differently from primary cells, we first designed experiments to determine both cell lines’ permissiveness and utility for mycobacterial infection. For this, cells were harvested at different timepoints after infection with different MOI of BCG^GFP^ and BCG^lux^. Here, we define permissiveness as the capacity of cell lines to get infected by mycobacteria without significant loss in cell viability. Utility, on the other hand, is the capability of cell lines to upregulate cell surface markers (Class I and II MHC molecules) relevant for conventional effector T cell targeting. Cells were collected at days 1, 2 and 3 to probe for cell viability (trypan blue exclusion method), mycobacterial infection (luciferase activity and BCG^GFP^-positive cells) and cell surface expression of MHC-I and -II molecules.

**Fig. 2:**
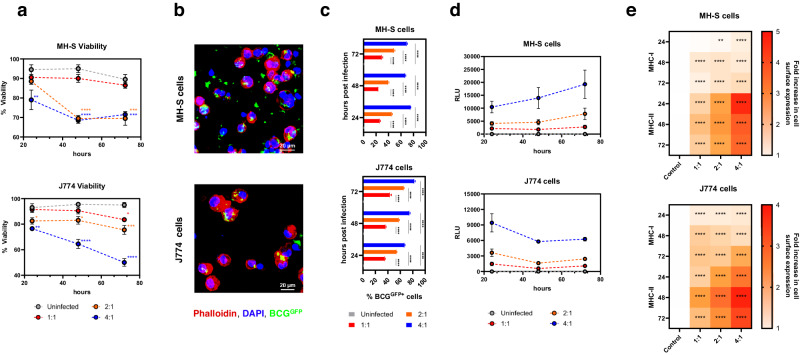
MH-S and J774 cell lines are permissive to mycobacterial infection and upregulate MHC Class I and II upon infection with BCG. **a** Line graphs showing viability and live cell counts of MH-S and J774 cells after infection with different MOI of BCG for 24, 48 and 72 h. **b** Representative confocal image showing BCG^GFP^ infection of MH-S and J774 cells after 24 h. **c** Bar graphs showing increasing frequencies of BCG^GFP^-positive MH-S and J774 cells with increasing MOI. **d** Line graphs showing viable mycobacteria after cell infection expressed as RLU. **e** Heatmap demonstrating the upregulation of MHC Class I and II relative to controls in MH-S and J774 cells after infection with different MOI of BCG for 24, 48 and 72 h. Error bars indicate SEM (*n* = 3). Statistical significance between groups was calculated using one-way ANOVA followed by Tukey’s multiple comparison test (**P < 0.01, ***P < 0.001, ****P < 0.0001).

First, we observed that both cell viability and live cell count were not significantly altered at low MOI (1:1) except for J774 cells at 72 h (*P* < 0.05) post infection; however, significantly reduced viability was seen at 2:1 (J774, *P* < 0.05; MH-S, *P* < 0.001) and 4:1 (J774 and MH-S, *P* < 0.001) MOI (Fig. 2a). The two cell lines were permissive to mycobacterial infection as shown by intracellular localisation of BCG using confocal imaging (Fig. 2b and Supplementary Fig. 3), increased frequencies of BCG^GFP^-positive cells (Fig. 2c) and viable mycobacteria (Fig. 2d) with increasing MOI. Cell surface expression of MHC-I and II was measured using flow cytometry. Overall, we observed a significantly greater increase in the cell surface expression of both MHC-I and MHC-II after BCG infection of cells (Fig. 2e). We also saw a trend towards higher MHC-I and MHC-II expression with increasing MOI.

Taken together, we observed that BCG infection of both cell lines resulted in variable changes in cell viability and cell count, MOI-dependent frequency of infected cells, and consistent upregulation of MHC-I and II molecules.

### Coculture of splenocytes from BALB/c mice and congenic cell lines results in restriction of BCG growth

**Fig. 3:**
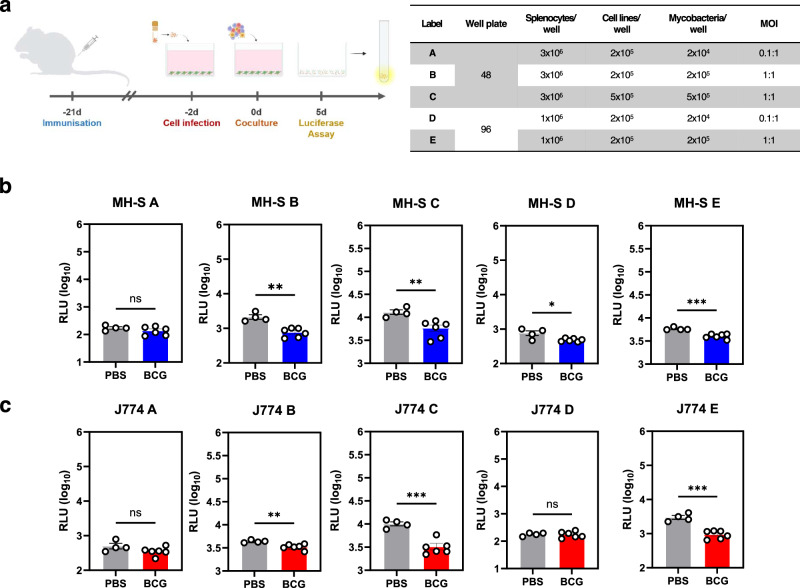
Coculture of mycobacteria-infected MH-S and J774 cell lines with splenocytes from BCG immunised BALB/c mice led to restriction of mycobacterial growth. **a** Cell lines were infected with BCG^lux^ under different MOI and cultured in either 96- or 48-well plates for 48 h. Splenocytes obtained from immunised and non-immunised mice were then cocultured with infected cell lines and incubated for 5 days. Cells were lysed and luciferase assay was done to quantify viable BCG per well. Bar graphs showing mycobacterial restriction, expressed as log_10_ RLU, for both **b** MH-S and **c** J774 cells. Error bars indicate SEM. Statistical significance between PBS and BCG groups was measured using unpaired *t* test (*P < 0.05, **P < 0.01, ***P < 0.001). Schematic in **a** created with BioRender.com.

Previous studies showed that coculture of either enriched T cell populations or splenocytes with infected cells led to mycobacterial killing in vitro^13–16^. To ascertain whether coculture of infected cell lines and splenocytes from immunised BALB/c mice can result in mycobacterial growth restriction, we obtained splenocytes from mice that were immunised subcutaneously with PBS or BCG, and cocultured single spleen suspensions with BCG-infected MH-S or J774 cells. To reduce the effect of dead cells in the assay, we opted to move forward with an MOI of 1:1 or lower for all coculture experiments. Here, we tested various conditions such as cell numbers, MOI (1:1 or lower), and well plates (96- and 48-wells) (Fig. 3a). We initially used a luciferase assay to optimise experimental conditions and obtain faster readouts. We demonstrated that both cell lines can be used to show restriction of BCG growth in vitro by splenocytes of immunised mice. Specifically, significant differences in mycobacterial growth restriction were consistently seen when ~2 × 10^5^ (MOI 1:1) (MH-S & J774 48B, *P* < 0.01; MH-S & J774 96B, *P* < 0.001) or ~5 × 10^5^ (MOI 1:1) (MH-S 48C, *P* < 0.01; J774 48C, *P* < 0.001) mycobacteria were added at day 0, but not when only ~2 × 10^4^ (MOI 0.1:1) mycobacteria were used except for MH-S 96A (*P* < 0.05). (Fig. 3b, c).

### Modified assay demonstrates mycobacterial restriction in C57Bl/6 mice

**Fig. 4:**
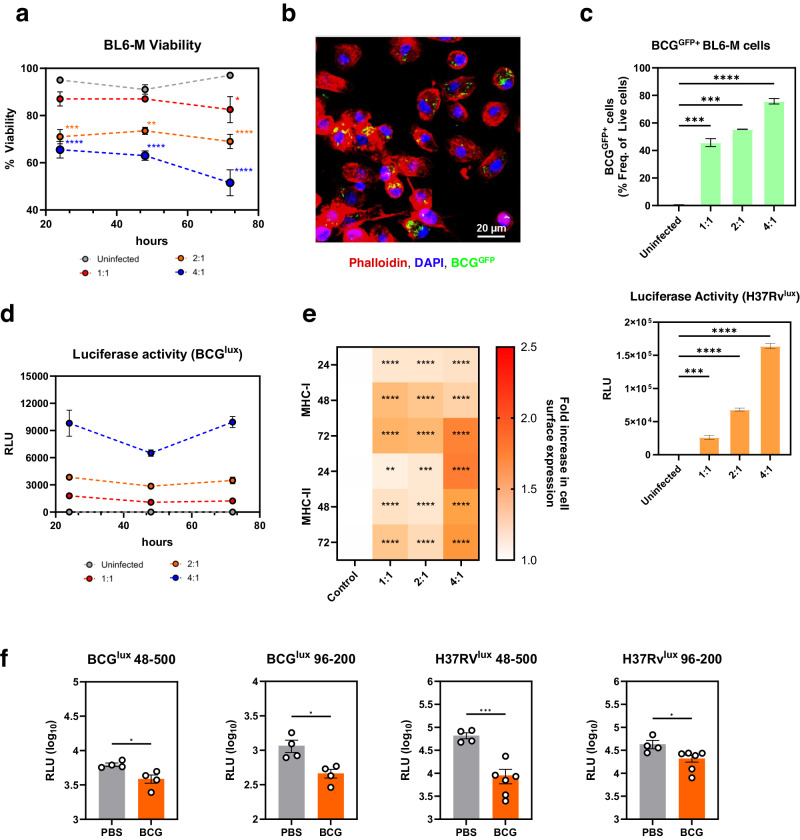
modMGIA can be utilised to demonstrate vaccine-induced mycobacterial killing in C57BL/6 mice. **a** Line graphs showing % viability and absolute live cell counts of BL/6-M cells after infection with different MOI of BCG for 24, 48 and 72 h. **b** Bar graphs showing infection burden of BL/6-M cell line with luciferase (BCG^lux^ and H37Rv^lux^) and fluorescent (BCG^GFP^) mycobacteria after 48 h. **c** Representative confocal image showing BCG^GFP^ infection of BL/6-M cells. **d** Line graph showing viable mycobacteria after cell infection expressed as RLU **e** Heatmap illustrating the upregulation of MHC Class I and II relative to control after infection with different MOI of BCG for 24, 48 and 72 h **f** Bar graphs show H37Rv and BCG luciferase activity after modMGIA. Error bars indicate SEM. Statistical significance between PBS and BCG groups was measured using unpaired *t* test (*P < 0.05, **P < 0.01, ***P < 0.001).

C57Bl/6 is another commonly used mouse strain in TB vaccine research. To determine the utility of the modified assay in this strain of mice, we obtained a C57Bl/6 background macrophage cell line, BL/6-M, and tested its permissiveness and utility for mycobacterial infection, and mycobacterial growth restriction after coculture with splenocytes from immunised mice. Similar to MH-S and J774 cells, we observed that BL/6-M cells were permissive to mycobacterial infection at all MOI used with significant reductions in viability and cell counts seen with higher MOI (MOI 2:1 – 24 h, *P* < 0.001; 48 h, *P* < 0.01; 72 h, *P* < 0.0001) (MOI 4:1 – 24 h, 48 & 72 h, *P* < 0.0001) (Fig. 4a). Confocal imaging also showed BCG^GFP^ internalisation by cells (Fig. 4b and Supplementary Fig. 3) while increasing MOI led to increased frequency of BCG^GFP^-infected cells, and viable BCG and H37Rv measured by luciferase assay (Fig. 4c and d). Cell surface expression of MHC-I and II was also significantly increased upon infection with different MOI of BCG, with highest expression observed at 72 h after infection (Fig. 4e). Interestingly, BL/6-M cells had higher cell surface expression of MHC-I compared to MHC-II in contrast to both MH-S and J774 cells.

To determine the suitability of infected BL/6-M cells for coculture with splenocytes, a similar experiment was performed wherein C57Bl/6 mice were immunised with PBS or BCG. As shown in Fig. 4E, we observed that the modified assay demonstrated BCG growth restriction in two different systems (96- and 48-well). More importantly, culture with a pathogenic Mtb strain, H37Rv, also led to significant growth restriction (96–200, *P* < 0.05; 48-500, *P* < 0.001) among mice immunised with BCG as compared to unimmunised animals (Fig. 4f).

Taken together, the modified assay can also be used to demonstrate mycobacterial restriction in C57Bl/6 strain of mice with the use of the BL/6-M macrophage cell line.

### Higher mycobacterial infection results in greater margin of difference between non-immunised and BCG immunised mice

**Fig. 5:**
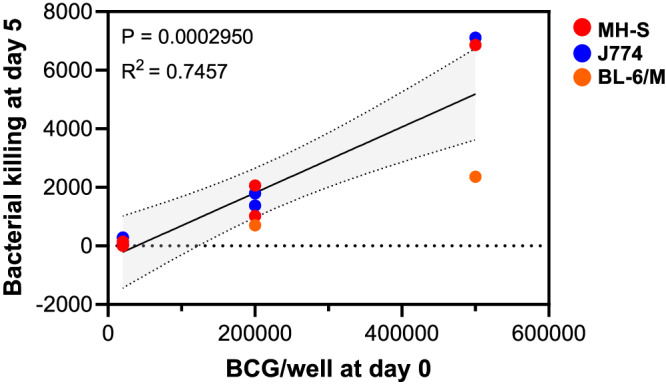
Initial bacterial load correlates with greater mycobacterial killing in modMGIA. Linear regression analysis to demonstrate correlation between the difference in bacterial load in PBS and BCG groups, and the initial bacterial infection of cell lines at day 0 in the modMGIA. Regression coefficient (*R*^2^) and *P* values are displayed in the figure.

We hypothesised that MGIA can be improved by increasing the number of mycobacterially infected target cells in the system. In our experiments with both BALB/c and C57Bl/6 mice, we demonstrated that coculture of splenocytes with infected cell lines resulted in the restriction of bacterial growth in BCG immunised animals. Plotting the CFU of BCG added to each well to infect cell lines regardless of cell number (X axis) and the difference in viable bacteria detected between the PBS and BCG groups (Y axis), we show that there is a strong correlation (*R*^2^ = 0.7457, *P* = 0.0002950) between the two parameters suggesting that increasing the initial mycobacterial burden in each well generates a higher margin of difference between unimmunised and immunised animals (Fig. 5). Interestingly, assays with the BALB/c background mice (MH-S and J774 cells; red and blue dots, respectively) appeared to have a greater margin of difference than C57BL/6 background mice (orange dots).

### Modified assay results in more mycobacterial killing than the conventional assay

**Fig. 6:**
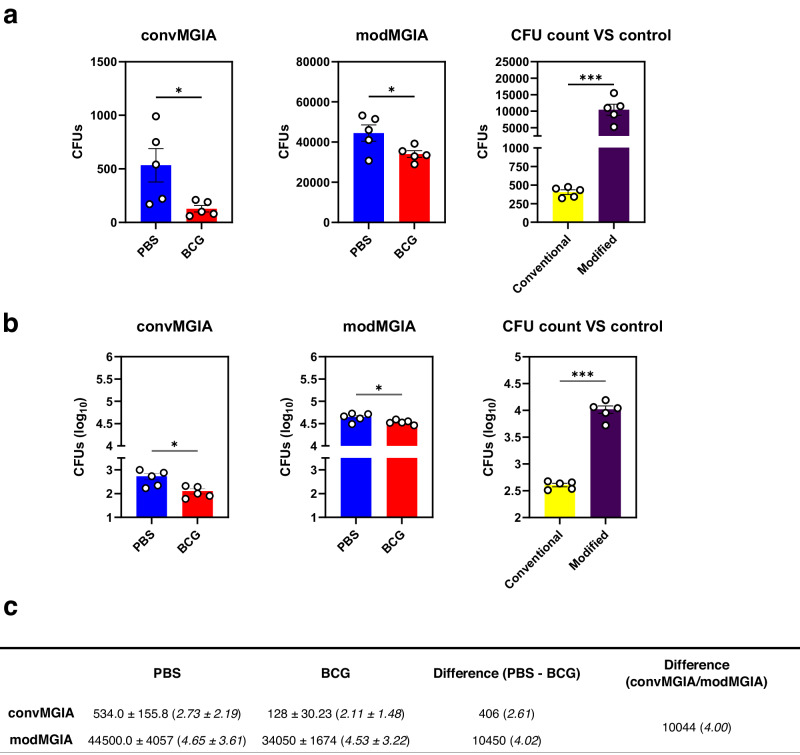
Modified assay results in significantly greater mycobacterial killing than the conventional assay. To quantitatively compare mycobacterial killing between conventional and modified assays, C57Bl/6 mice were immunised with BCG or given PBS as control. Spleens were harvested 21 days after immunisation and were used for conventional and modified MGIA. Bar graphs showing bacterial loads and level of mycobacterial killing relative to the control plotted in **a** linear or **b** logarithmic scale. **c** summary table showing values obtained from PBS and BCG groups for conventional and modified assays, and the difference in quantified bacteria between the two assays Error bars indicate SEM. Statistical significance between PBS and BCG groups was measured using unpaired *t* test (*P < 0.05, ***P < 0.001).

Next, we hypothesised that increasing the number of mycobacteria-infected cells in the culture system could lead to better performance of the assay since more target cells can increase the margin of difference between unimmunised and immunised groups. As the conventional assay has fewer target cells, we wanted to show that the modified assay can demonstrate greater killing in terms of number of mycobacteria killed per set quantity of splenocytes in the culture system. To test this, we performed both conventional and modified assays using splenocytes obtained from PBS or BCG immunised mice. We then quantified viable mycobacteria using CFU-based methods. As shown in Fig. 6a–c, modMGIA detected a considerably higher number of infected cells than convMGIA, with PBS group detecting 534 ± 155.8 (Log_10_2.73+/12.19) in convMGIA and 44500 ± 4057 (Log_10_ 4.65 ± 3.61) CFU using modMGIA, an 83-fold increase. When we compared immunised (BCG) and nonimmunized (PBS) groups, we observed a significant reduction in viable mycobacteria for both the convMGIA (*P* < 0.05) and modMGIA (*P* < 0.05). These differences, when expressed as relative reduction of CFUs on a logarithmic scale, do not appear to distinguish the size of the effect between the two methods, which however, becomes apparent when the total number of infected cells is considered. Thus, when comparing the conventional and the modified assay in terms of the total number of target cells killed by the same number of splenocytes by subtracting mean CFUs for BCG group from those for the PBS group, we demonstrate that the modified assay results in significantly greater mycobacterial killing (*P* < 0.001) as compared to the conventional method (Fig. 6c). Notably, the same number of splenocytes killed 2.61 log_10_ CFU (406 mycobacteria) in the conventional assay and 4.02 log_10_ CFU (10450 mycobacteria) in the modified assay, a 26-fold increase. These measurements demonstrate that the modMGIA gives a greater scope for assessing the functionality of the immune cells, once the restriction of the low number of permissive target cells in the assay is lifted.

### Mycobacterial killing of BCG but not H37Rv correlates with secreted IFNγ and TNFα in the modified assay

To probe whether a correlation exists between mycobacterial killing and secreted IFNγ and TNFα, culture supernatants were obtained at the end of the assay prior to measurement of viable mycobacteria in each well. Doing so allowed us to plot the levels of secreted cytokines in the supernatant against the viable mycobacteria measured from each well.

**Fig. 7:**
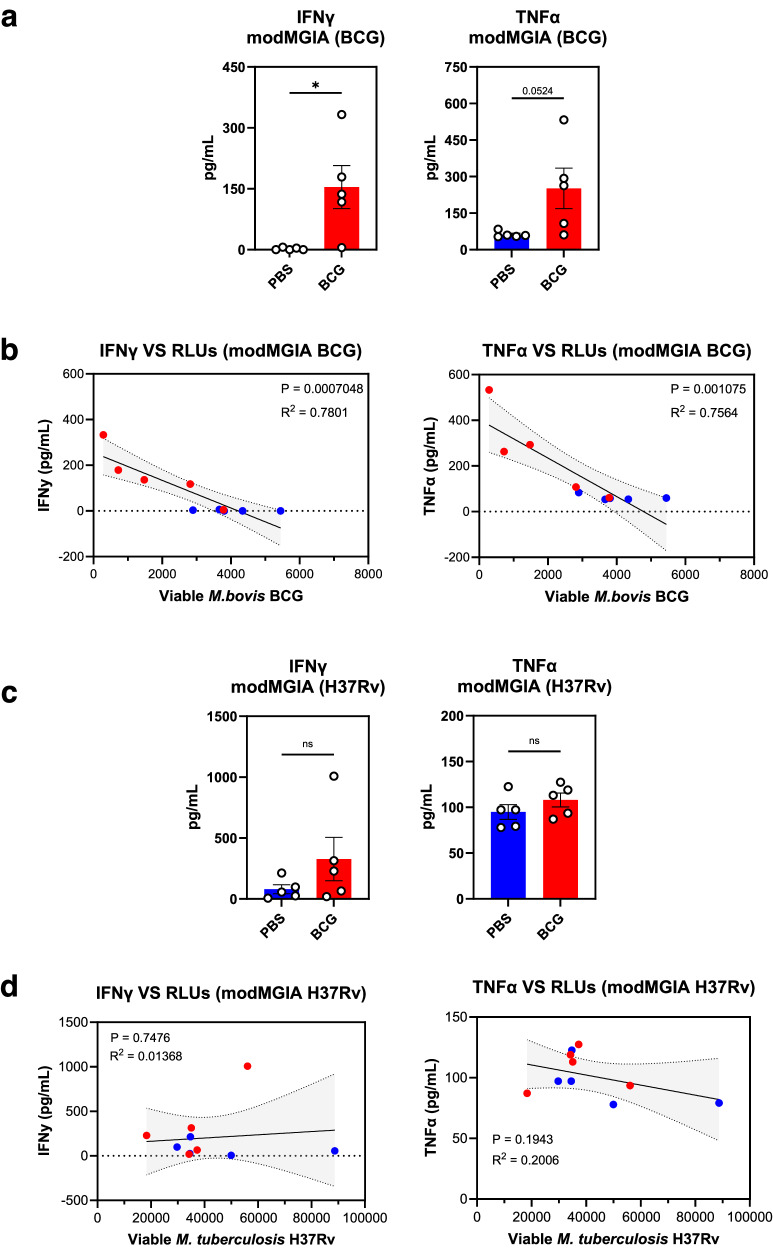
In vitro mycobacterial killing of BCG but not H37Rv seen in the modMGIA correlates with secreted TNFα and IFNγ. modMGIA was done by coculturing 5 × 10^5^ infected cells (MOI = 1:1) and 3 × 10^6^ splenocytes from unimmunised and immunised mice. To probe for the role of Th1 cytokines, IFNγ and TNFα, cell culture supernatants from the modified assay were collected at the end of the timepoint followed by cytokine measurement by ELISA. **a** Secreted IFNγ and TNFα after 120 h of coculture of BCG-infected cells and splenocytes from PBS and BCG groups. **b** Correlation analysis of secreted cytokines (IFNγ and TNFα) *vs* viable bacteria (RLU) using linear regression model. **c** Secreted IFNγ and TNFα after 120 h of coculture between H37Rv infected cells and splenocytes from PBS and BCG groups. **d** Correlation analysis between secreted cytokine (IFNγ and TNFα) *vs* viable bacteria (RLU) using linear regression model. Error bars indicate SEM. Statistical significance between PBS and BCG groups was measured using unpaired *t* test (*P < 0.05). Regression coefficient (*R*^2^) and *P* values are displayed in the figure.

First, we demonstrated that modMGIA using BCG-infected cells, splenocytes from BCG immunised mice had greater IFNγ (*P* < 0.05) and TNFα (*P* = 0.0524) secretion than unimmunised animals (Fig. 7a). Secreted levels of both cytokines strongly correlated (*R*^2^ = 0.7801, IFNγ and *R*^2^ = 0.7564, TNFα) with viable BCG in the wells (Fig. 7b). Interestingly, we did not see the same trend when H37Rv was used to infect cells for the modified assay. Although we did observe a noticeable increase in levels of IFNγ in the BCG group, only a marginal increase was seen for secreted TNFα (Fig. 7c). In addition, the secreted cytokines did not correlate with viable H37Rv (Fig. 7d). The secreted cytokines in the conventional assay did not follow the same trend as the modified assay and no correlation between secreted TNFα, and viable bacteria was seen (Supplementary Fig. 4a, b).

Overall, we saw a trend towards higher secreted IFNγ and TNFα in the modified assay when BCG was used to infect the cell line. This was not seen when H37Rv was used to infect cell lines suggesting that a different mechanism in addition to the functions of the cytokines we measured may be involved in the killing of H37Rv.

### modMGIA shows consistent inter-assay readings between luciferase- and CFU-based methods to quantify live mycobacteria

**Fig. 8:**
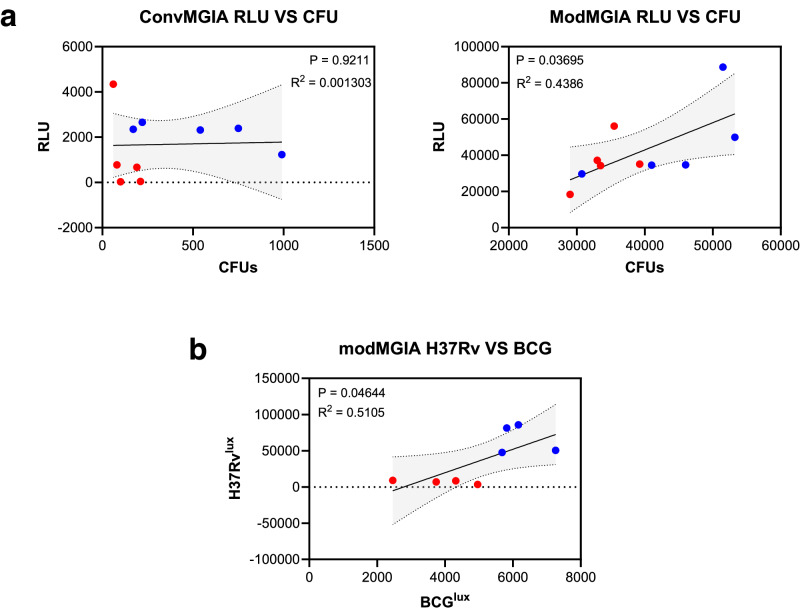
Modified assay results in consistent results between luciferase assay and CFU method. **a** To determine inter-assay consistency between the luciferase assay and plating method, we performed linear regression analysis wherein RLU and CFU values in individual samples from both the PBS and BCG group were plotted on the y and x axes, respectively. **b** Linear regression analysis plotting data obtained from modMGIA that used either BCG or H37Rv to infect BL6-M cells. Regression coefficient (*R*^2^) and *P* values are displayed in each panel.

We also wanted to probe whether conventional and modified assays generate consistent inter-assay results with the methods we used to quantify mycobacteria: luciferase assay and the CFU method. For this, we plotted the individual results obtained from the luciferase assay (RLU) on the Y axis, and the corresponding results obtained for the CFU method on the Y axis (Fig. 8a). Our analysis revealed that inter-assay consistency was only seen with modified assay (*R*^2^ = 0.4386, *P* = 0.03695). Next, the inter-assay consistency between modMGIA that used BCG or H37Rv to infect the cell lines was determined by plotting individual results obtained from modMGIA-H37Rv on the Y axis and modMGIA-BCG on the X axis. It was observed that a mild correlation (*R*^2^ = 0.5105, *P* = 0.04644) exists between the values obtained from the two assays demonstrating that both laboratory strains of mycobacteria can be used for the modified assay to demonstrate mycobacterial killing (Fig. 7b). Together, we showed that using modMGIA generates consistent results regardless of the method to quantify viable mycobacteria.

Intra-assay and inter-assay coefficients of variation were also assessed to determine the reproducibility of the assay (Supplementary Table 1). First, we observed that CV in the BCG group (1.55–8.21%) was generally higher than the CV of the PBS group (0.68-4.83%). Intra-assay variability ranged from 3.01–4.94%, 1.1–4.6% and 1.4–5.31% for experiments with MH-S, J774 and BL-6M cell, respectively. Finally, inter-assay variability across all modMGIA experiments was 3.41%. Interestingly, the modified assay had a lower CV than the conventional assay.

## Discussion

The conventional method of MGIA is performed by coculture of splenocytes and virulent or avirulent mycobacteria^5,6^. We hypothesised that increasing the number of mycobacteria-infected target cells in the assay could lead to increased discriminatory capacity between unimmunised and immunised populations of mice since the mixed population of cells in spleens may lead to restrictively low number of infected target cells. As previously shown, coculture of primary macrophages obtained from either the peritoneum or bone marrow of mice led to mycobacterial killing when cocultured with splenocytes or enriched CD4 or CD8 T cells^13–15^. As we wanted to establish a protocol that reduces the reliance on animals, we tested coculture of cell lines with splenocytes from immunised BALB/c or C57Bl/6 mice. Herein, we have established a more discriminatory functional assay that is also robust and reproducible in two commonly used strains of mice.

Since this functional assay is reliant on the interaction of pMHC expressed by infected cells and TCR of primed CD4 and CD8 T cells, it was thus important to demonstrate permissiveness of cell lines to mycobacterial infection, as well as upregulation of cell surface MHC molecules post infection. Previous studies have shown that cell lines may behave differently from primary cells upon infection with mycobacteria^26^ and this can be attributed to the cell lines’ phenotypic or genetic changes^26,27^. Here, we used three different cell lines: two on BALB/c background (alveolar macrophage [MH-S] and peritoneal macrophage [J774]) and one on C57Bl/6 background (bone marrow-derived macrophage [BL/6-M]). MH-S and J774 were previously used for macrophage-Mtb interaction studies^26,28^. On the other hand, a BL/6-M cell line, BMA3.1A7, was used to study the consequence of mycobacterial infection in the permeability of phagosomes^29^. Previous studies comparing the impact of mycobacterial infection between cell lines and primary cells revealed mixed results. One study demonstrated that MH-S cells had a similar phagocytic capacity for Mtb to primary alveolar macrophages^28^. In contrast, comparative transcriptomic profiling of J774 cells and primary bone marrow-derived macrophages post Mtb infection revealed earlier changes in pathways attributed to enhanced inflammatory responses and bacterial control in primary BMDMs^26^. In our experiments, we showed that all three cell lines can internalise mycobacteria, as well as upregulate cell surface expression of MHC-I and II molecules, albeit at different levels of permissiveness.

In our modified MGIA, we adapted cell culture conditions that previously demonstrated increased viability of splenocytes^6^. Specifically, we performed static cocultures of infected cell lines and splenocytes in flat-bottom well plates using enriched medium. Previous iterations of the assay cultured murine cells in rotating tubes which led to significant reduction in the viability of splenocytes, thus decreasing the reliability of the assay results^6^.

Apart from culture conditions, we also assessed several assay parameters such as initial mycobacterial infection per well, ratio of splenocytes to cell lines and the type of multi-well plates used. Previously, it was shown that culture of cells for MGIA can be done in a 24-, 48-, or 96-well plates^13–16^. Meanwhile, variations in MOI for coculture assays were also seen in different studies ranging from 1:100 (i.e. 1 bacterium for 100 cells)^13,14^ or 0.2, 0.6, 0.8 or 2 Mtb per cell^15,16^. As we did not find any prior evidence for the impact of MOI on cell viability and cell surface expression of MHC, we opted to test several MOIs using BCG as the surrogate mycobacteria. Our analysis revealed that a greater initial mycobacterial infection burden results in a greater magnitude of difference in viability and MHC expression between the unimmunised and BCG immunised groups.

MHC-I and -II drive immunity against mycobacteria through priming of naïve T cells and subsequent recognition of infected cells by conventional CD8+ and CD4 + T cells, respectively^30–32^. It is thus crucial that MHC molecules are recognised by T cells in the modified assay through MHC, so that effector T cells can drive intracellular killing or mycobacteria. Here, we demonstrated that infection with BCG upregulated the cell surface expression of MHC-I and II in all three cell lines. Upon recognition of pMHC complex on the surface of infected cells, effector T cells drive intracellular killing of mycobacteria by several means^33^. One mechanism by which effector T cells can drive intracellular killing of mycobacteria within macrophages is through secreted IFNγ and TNFα. These cytokines were shown to drive killing of mycobacteria within matured phagosomes by driving autophagy in macrophages^34^, as well as bacterial killing through apoptosis of infected macrophages^35,36^.

The primary aim of this study was to determine whether using a modified assay could significantly increase the reliability of results, in part due to increased discriminatory capacity between cell populations obtained from non-immune (PBS group) and immune (BCG immunised) groups of mice. In previous studies using the convMGIA, a difference between unimmunised and BCG immunised groups varied from 0.3 log_10_^6^ to 0.93–1.12 log_10_^5^ using splenocytes as samples, and 0.49–0.60 log_10_ when lungs were used^5^. In this study, we observed a 0.58 log_10_ difference between the BCG and PBS groups, which was consistent with the results obtained by Jensen et al.^6^. Comparing this to the modified assay, we observed that the relative reduction in mycobacterial load was 0.11 log_10_ and this was due to the very high initial bacterial burden added to each well. However, when expressed as the difference between the PBS and BCG groups in terms of bacterial load in the system, we observed that the modMGIA actually resulted in as much as 26 times greater bacterial killing than the convMGIA (2.61 VS 4.02 log_10_ CFU killed). We argue that this makes modMGIA a more useful assay than convMGIA since having greater number of mycobacteria that can be killed can better discriminate the effector T cell response generated by vaccine candidates.

As many of the published articles on MGIA utilised the BACTEC MGIT system, which is a more sensitive liquid culture system to quantify mycobacteria in samples, the modified assay could also be adapted for that protocol to further increase sensitivity.

Potential problems that could be encountered with modMGIA and proposed mitigation measures are shown in Supplementary Table 2. These include lack of access to the cell lines used in the study, inconsistency of results, lack of containment level 3 (CL3) facilities, no access to microbiological plating, and difficulty in translation to human samples.

Although we did not test any vaccine candidate other than BCG in this study, we have shown previously that using this modified assay generates results that were correlated to lung bacterial load obtained in a mouse challenge study^37^. However, additional parallel experiments wherein the result of the modified assay are compared for multiple vaccine candidates and correlated with tissue bacterial burden in animal challenge experiments will further strengthen evidence as to whether the assay is more predictive of vaccine protection conferred in vivo than the conventional MGIA assay. We furthermore predict that its higher discriminatory ability will be particularly useful when screening and ranking vaccines in the early stages of development that otherwise may be performing similarly or only marginally differently in the conventional MGIA.

## Methods

### Ethics statement

Animals were used in compliance with the Animal (Scientific Procedures) Act 1986, and with approval from St. George’s University of London (SGUL), London School of Hygiene and Tropical Medicine (LSHTM) and University of Leicester (UoL) Ethics approval under Home Office animal project licenses 70/7490 and P6DCE1A76 was obtained. The study was carried out in compliance with the ARRIVE guidelines.

### Mice and immunisations

All mice used in the experiments were kept under specific pathogen free (SPF) conditions at the Biological Resource Facility at SGUL, Biological Services Facility of LSHTM, and the preclinical resource facility of UoL. All animals were fed with a standard rodent diet and provided with water *ad libitum*. For LSHTM animal experiments, 6- to 8-week-old BALB/c mice were purchased from Charles River, randomly allocated in groups of 5–6 per cage and acclimatised for 7 days. For SGUL and UoL experiments, BALB/c and C67BL/6 mice, respectively, were sourced from the in-house breeding facility, age-matched and randomly allocated in groups of five to six animals per each cage. Mice were immunised with 100 µL sterile Dulbeco’s phosphate buffered saline (DPBS) or BCG Pasteur (5 × 10^5^ CFUs/mouse) subcutaneously at the base of the tail. After 3 or 7 weeks, euthanasia by cervical dislocation was done and spleens were collected from each mouse.

### Culture and use of laboratory strains of mycobacteria

Mtb H37Rv, Mtb H37Rv-luciferase (Mtb^lux^), BCG Pasteur (BCG^WT^), BCG-luciferase (BCG^lux^) and BCG expressing green fluorescent protein (BCG^GFP^) were cultures maintained at the Institute for Infection and Immunity, St. George’s, University of London. H37Rv and BCGW^WT^ were propagated in Difco™ Middlebrook 7H9 Broth (BD, 271391) supplemented with 10% v/v Middlebrook 7H10 oleic acid, albumin, dextrose, and catalase (OADC) (USBiological Life Sciences, M3895-01) and 0.5% v/v glycerol (VWR Chemicals, 56-81-5). Culture media for Mtb^lux^, BCG^GFP^ and BCG^lux^ were supplemented with 50 µg/mL hygromycin B (ChemCruz^®^, SC-29067) as a selection marker and 0.05% tyloxapol (Sigma, T0307-10G) as detergent to prevent clumping. All mycobacterial cultures were propagated for up to 3 weeks. Frozen stocks were made with sterile deionised distilled water with 10% glycerol kept at −80 °C or −196 °C. The percentage of GFP-expressing BCG in samples was >90% as determined by flow cytometry (data not shown).

To quantify colony-forming units (CFU) in samples, serial dilutions of bacteria were plated on solid media comprising of BBL™ Seven H11 agar base (BD, 212203) supplemented with OADC and Mast^®^ Selectatab (Mast group, MS24). Plates were kept in a sealed bag and left in an incubator set to 37 °C for around 3 weeks. Contamination was monitored by assessing for growth of contaminants for 48–72 h after initial seeding of cultures. All experiments with BCG were performed in Class II biosafety cabinets and experiments with Mtb in Class I biosafety cabinets housed in the CL3 suite of St. George’s University of London.

### Luciferase assay

Luciferase assay was done to quantify viable bacteria as previously described^38^. Briefly, cell lysates were added to tubes (Greiner Bio-One, 115101) with 1 mL of 0.1% *n*-decyl aldehyde (Decanal) (Sigma-Aldrich, W236217), prepared by doing a 1 in 10 (v/v) dilution of 1% Decanal in PBS. Bioluminescence was measured using a Junior LB 9509 Portable Luminometer (Berthold Technologies GmbH & Co.) set to measure luminescence for 30 s. Data are expressed as relative light units (RLU).

### Murine cell lines and cell culture

J774^39^ (BALB/c peritoneal monocyte cell line) and MH-S^40^ (BALB/c alveolar macrophage cell line) were obtained from ATCC while the BL/6-M^41^ (C57Bl/6 bone marrow-derived macrophage cell line) cell line was obtained from Kerafast® (ENH166-FP). RPMI (Sigma, R0833) and DMEM (Sigma, D6546) were supplemented with 10% Foetal Bovine Serum (FBS,) (Sigma-Aldrich, F9665), 5mM L-Glutamine (Sigma, G7513), 100 U/mL Penicillin & 100 µg/mL Streptomycin (Sigma, P4333), 50 µM β-mercaptoethanol (Sigma, M3148), and 10 mM (4-(2-hydroxyethyl)-1-piperazineethanosulfonic acid (HEPES) buffer (Gibco, 15630-056), and used in all assays, except for omission of antibiotics in infection assays. Supplemented DMEM and RPMI-1640 are herein referred to as D10 and R10, respectively. MH-S cells were maintained in R10 (Gibco) while J774 and BL/6-M cells were initially cultured in D10 but were conditioned to be cultured in R10 media since splenocytes used for coculture experiments required the use of R10. There was no significant difference in the viability of J774 and BL/6-M cells after culture in R10 medium. For infection assays, cells were detached by first removing media, then gently washing the cells twice with sterile DPBS (Sigma, D8547). Afterwards, cells were incubated for 5 mins in cell dissociation buffer (EMD Millipore Corp USA, W4502) and harvested by washing the plate with complete medium. After centrifugation at 300 rcf for 5 mins, cells were resuspended in complete medium and counted using an automated cell counter (TC20™, Bio-Rad Laboratories, Inc.) using Trypan Blue exclusion method. All cell cultures were maintained in a humidified CO2 incubator (5% CO_2_ and 37° C).

### Infection of cell lines BCG^l^^ux^, BCG^GFP^ and Mtb^lux^

MH-S, J774 and BL/6-M cells were seeded in 96- (Corning, 353072) or 48- (Corning, 3548) well plates at a density of 500,000 cells per well. After leaving the cells to settle for 2 h, cells were infected with mycobacteria at different multiplicities of infection (MOI): 1:1, 2:1, and 4:1. Infected cell lines were harvested from multi-well plates by aspirating culture medium, then adding cell dissociation buffer. After 5 mins in the CO2 incubator, fresh medium was added to each well and cells were detached from the bottom of the wells by vigorous pipetting. Cells were transferred to 96-well U bottom plates and washed thrice with sterile PBS prior to FACS staining and analysis. For luciferase assays, cells were directly lysed with 200 µL sterile water (Sigma, 3864580) after aspiration of culture medium.

### Mouse splenocyte isolation and culture

Aseptically collected spleens were placed in tubes with MACS^®^ tissue storage solution (Miltenyi Biotec, 130-100-008) and maintained on ice to keep the integrity of the organs prior to processing. Single-cell suspensions were obtained by mechanical maceration of the spleen with a syringe barrel through a 70 µm cell strainer (Falcon, 352350) in a 50 mL Falcon tube. The cell strainer was rinsed with 10 mL complete RPMI-1640, then tubes were centrifuged at 300×*g* for 5 mins in a cold centrifuge set to 4 °C. After discarding the supernatant, red blood cells (RBCs) were lysed by the addition of 2 mL ACK lysis buffer (Gibco, A10492-01), and incubation at room temperature for 2 mins. Lysis was arrested through the addition of 20 mL R10 medium and tubes were again centrifuged at 300×*g* for 5 mins. Pelleted cells were resuspended in complete RPMI-1640 medium and counted by Trypan Blue exclusion method. All experiments with spleen cells were carried out in complete R10 unless otherwise indicated.

### Infection of splenocytes with BCG^GFP^

One million spleen cells were seeded in 96 flat-bottom well plates. MOIs were either 0.5, 1, or 2. BCG^GFP^ infection of splenocytes was carried out for 24 h. Wells were washed thrice with 200 µL sterile DPBS prior to FACS staining and analysis.

### Conventional and modified mycobacterial growth inhibition assay

A modMGIA was used to assess the capacity of mouse splenocytes for in vitro killing of BCG or Mtb (Supplementary Fig. 1). Initial experiments using BALB/c mice and two congenic cell lines (MH-S and J774) were done to test different conditions including cell density in different well plates (96- and 48-well plates), and ratio of splenocytes to cells infected with mycobacteria. For these experiments, cell lines were infected as above mentioned (MOI 0.1 or 1:1) for 48 h before coculture, culture medium was removed and then replenished with fresh medium (100 µL for 96-well plate and 700 µL for 48 well plates). This was followed by the addition of splenocytes to designated wells (100 µL of 1 × 10^6^ for 96-well plates and 300 µL of 3 × 10^6^ for 48 well plates). For conventional MGIA, 3 × 10^6^ splenocytes plated in 48 well plates were directly infected with 500 CFUs of Mtb. Both conventional and modified MGIA cultures were left for 120 h, followed by aspiration of the media, and cell lysis with sterile distilled water. Cell lysates were used for CFU- or luciferase-based assays to quantify viable mycobacteria. In some experiments, aspirated media were collected for secreted cytokine analysis.

### Cell surface staining and flow cytometry analysis

Splenocytes or cell lines were added to 96-well U-bottom plates and centrifuged at 300×*g* for 5 min. Cells were then washed with sterile DPBS thrice, then stained using a cocktail of antibodies (1:200 PE anti-mouse H-2, Clone M1/42, BioLegend^®^ 125506; and 1:200 Brilliant Violet 510^TM^ anti-mouse I-A/I-E, Clone M5/114.15.2, BioLegend^®^ 107635), 1:500 eBioscience^TM^ fixable viability dye eFluor^TM^ 780 (Invitrogen,65-0865-14), and 1:250 mouse Fc block (TruStain fcX™ [anti-mouse CD16/32]) (BioLegend^®^, 422302) for 30 min at 4 °C. Afterwards, cells were washed three times then resuspended in sterile 1X PBS prior to FACS acquisition using CytoFlex (Beckman Coulter). For all experiments, at least 20,000 live events were obtained per sample. Flow cytometry results were analysed using FlowJo^TM^ v.10.8 (BD Life Sciences). Representative gating strategy and histograms are shown in Supplementary Fig. 2.

### Cytokine ELISA using cell culture supernatants

Commercially available kits (all from Invitrogen) were used to quantify IFNγ (88-7314-88) and TNFα (88-7324-88) from culture supernatants. Plates were coated with 100 µL capture antibody in PBS overnight at 4 °C. After three wash steps with PBS + 0.05% Tween-20 (PBS-T), wells were blocked with 200 µL blocking buffer for 1 h at room temperature. Wells were then washed three times, then 100 µL of neat supernatants or serial dilutions of the standards were added to wells. After 2 h of incubation at room temperature, wells were washed five times with PBS-T, followed by the addition of 100 µL of detection antibody. Plates were incubated at room temperature for an hour and washed five times with PBS-T. Wells were then incubated for 1 hr at room temperature with 100 µL of streptavidin-HRP. After seven washes with PBS-T, TMB substrate was added to each well. Plates were left to develop for 15 mins, followed by the addition of stop solution (0.16 M H_2_SO_4_, In-house). The absorbance reading for each plate was obtained at 450 nm with a correction of 540 nm using a plate reader (Tecan, UK). Absorbance readings from blank wells were subtracted from all wells with samples or standard.

### Confocal microscopy

To visualise internalisation of BCG^GFP^ by cells, 50,000 cells were added to 96-well clear bottom black plates (Corning Inc., 3603). After 2 h, BCG^GFP^ was added (MOI = 4) to cells followed by incubation for 24 h. Wells were gently washed with DPBS thrice, followed by cold fixation and permeabilization for 15 min using IC fixation buffer (Invitrogen, 00-822-49). Wells were then gently washed twice with 1X permeabilization buffer (Invitrogen, 00-8333-56), followed by staining for F-actin using Phalloidin-iFluor647 (1:2000) (Abcam, ab176759) for 45 min in 4 °C. After three washes with 1X permeabilization buffer, nuclear counterstaining for 5 min was done using 4’,6-diamidino-2-phenylindole, dihydrochloride (DAPI) (1:1000) (Thermo Fisher Scientific, D1306). Stained cells were finally resuspended in DPBS, followed by image acquisition using Nikon A1R confocal microscope and NIS Elements software. Images were generated using NIS Viewer v 5.21.00 (Nikon, UK).

### Statistical analysis

All statistical analyses were performed in GraphPad Prism Version 9.2.0 (La Jolla, CA). Where applicable, unpaired t-test, one-way ANOVA followed by Dunnett’s or Tukey’s test for multiple comparisons, or two-way ANOVA followed by Tukey’s range test as post-hoc analysis was done. All data shown are expressed as mean ± SEM. *P < 0.05, **P < 0.01, ***P < 0.001 and ****P < 0.0001 were considered significant. Intra-assay and inter-assay coefficient of variations (CV) were calculated using the formula: (standard deviation/mean × 100). Intra-assay precision represents the means between CV of PBS and BCG groups, and inter-assay precision is the mean CV of results from modMGIA.

### Supplementary information

Supplementary Material

## Data Availability

The datasets used and/or analysed during the current study are available from the corresponding author on reasonable request.
