# Supplementary material for: A modified mycobacterial growth inhibition assay for the functional assessment of vaccine-mediated immunity
Source: NPJ Vaccines. 2024 Jul 2;9:123. doi: 10.1038/s41541-024-00906-z (PMC11219912; doi:10.1038/s41541-024-00906-z)
Supplement: Supplementary file 1 — Supplementary Material [file 41541_2024_906_MOESM1_ESM.pdf]

Supplementary Files

A modified mycobacterial growth inhibition assay for the functional assessment of vaccine-mediated immunity

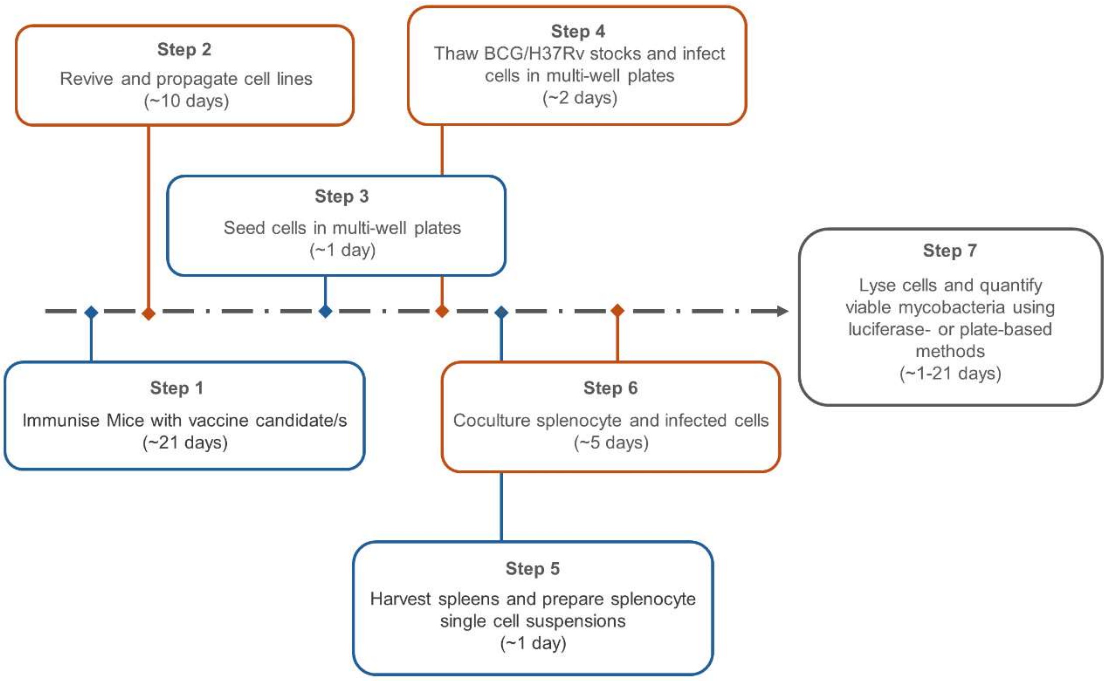

Supplementary Figure 1. Schematic representation of all the necessary steps and approximate time needed for each part of the modified MGIA.

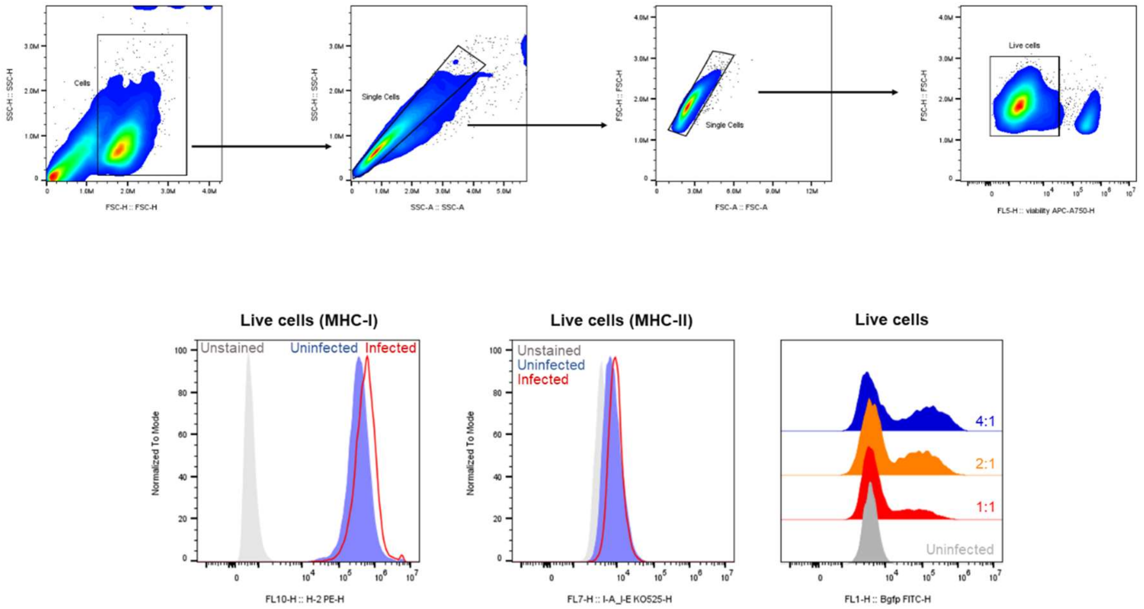

Supplementary Figure 2. Gating strategy and representative histogram overlays for measurement of BCG^{GFP}+ cells and cell surface expression of MHC I and II molecules in J774, MH-S and BL/6-M cell lines. Cell lines were plated in 96- or 48-well plates, and left for 24, 48 or 72 hours uninfected or infected with BCG at different MOI. Afterwards, cells were harvested, and surface stained for MHC-I and MHC-II molecules. Following FACs acquisition, data were analysed, and MFI was quantified. The % frequency of BCG^{GFP}+ cells were also determined.

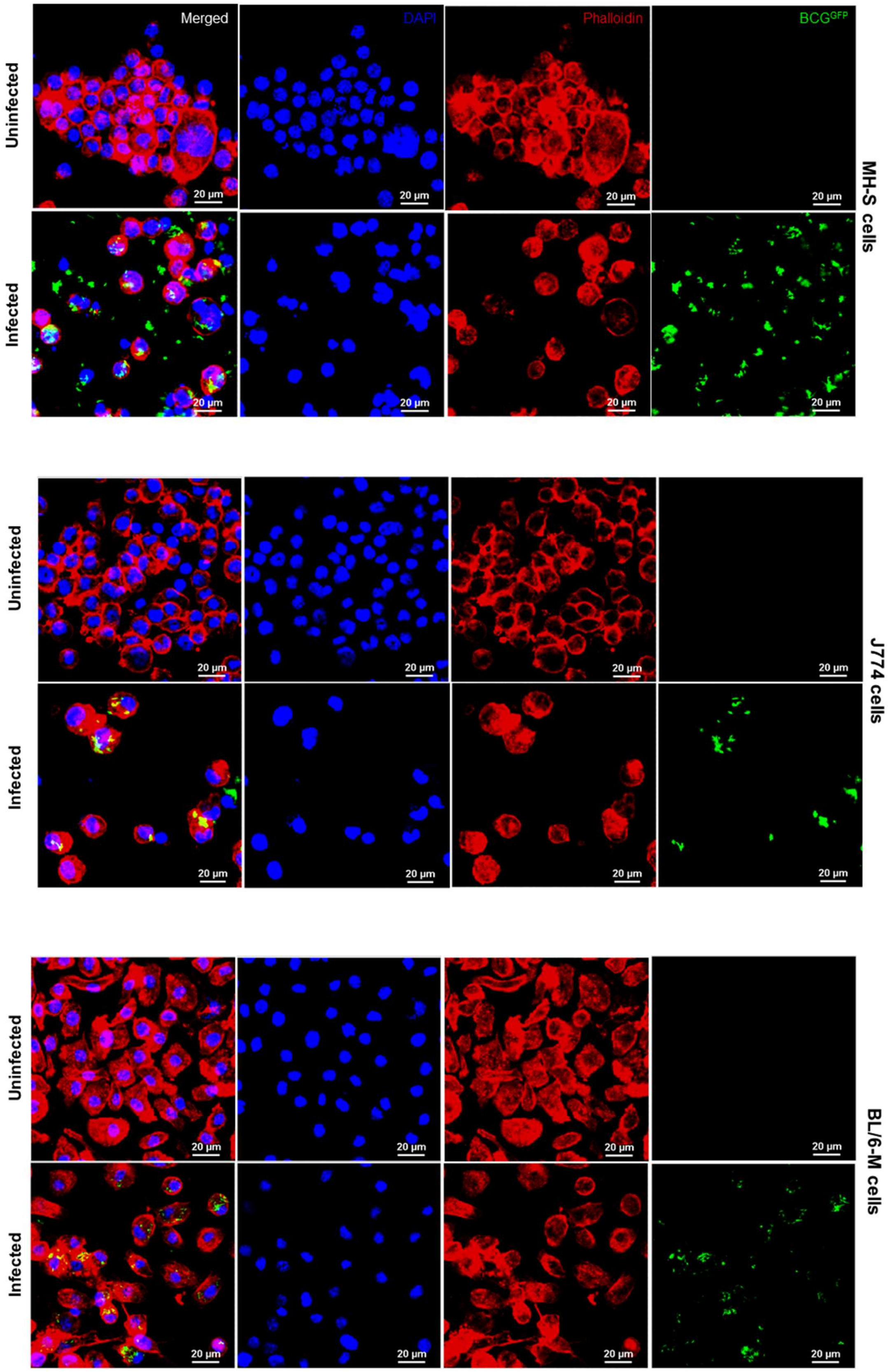

Supplementary Figure 3. Representative confocal images following BCG^{GFP} infection of MH-S, J774 and BL/6-M cell lines. Cell lines were plated in a 96-well black plates and

were left uninfected or infected with BCG^{GFP} (MOI = 4:1) for 24 hrs. Cells were then stained for F-actin (red) and nucleus (blue) using phalloidin and DAPI, respectively.

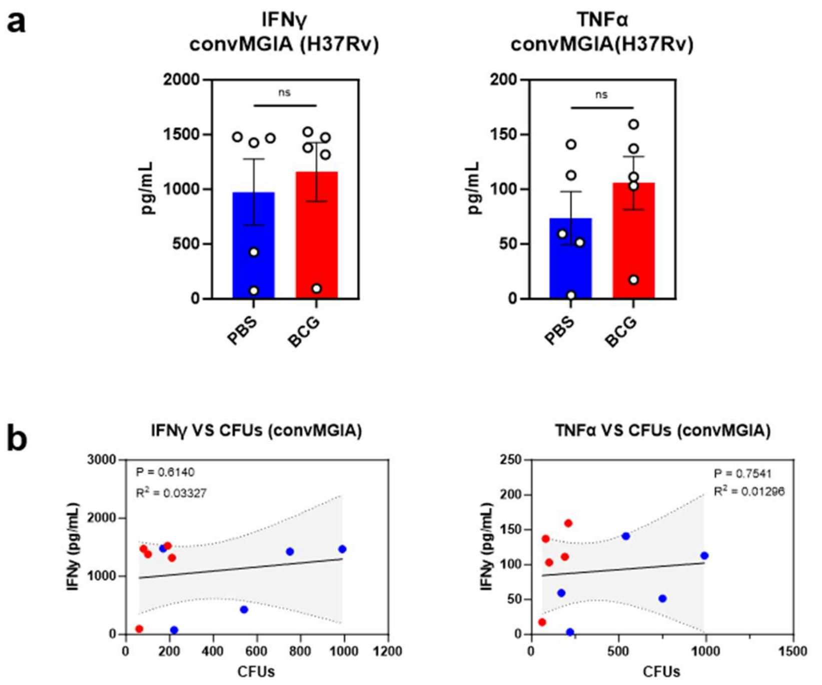

Supplementary Figure 4. Secreted IFNγ and TNFα in conventional MGIA. Splenocytes from non-immunised and immunised mice were plated (3x10^6/well) in 48 well plates and infected with 500 CFUs H37Rv. Culture supernatants were obtained from each well after 120 hrs to measure IFNγ and TNFα through ELISA. **a** Bar graph showing secreted IFNγ and TNFα. **b** Correlation analysis between secreted cytokine (IFNγ and TNFα) VS viable bacteria (CFU) using linear regression model. Error bars indicate SEM. Statistical significance between PBS and BCG groups was measured using unpaired t test.

Supplementary Table 1. Calculated coefficient of variations in the assay

Figure in manuscript	Label	CV (%) (PBS)	CV (%) (BCG)	Intra-assay CV (%)	Inter-assay CV (%)
Figure 3B	MH-S A	3.35	6.55	4.95	3.41
	MH-S B	3.19	4.18	3.68	
	MH-S C	2.09	4.58	3.33	
	MH-S D	4.47	1.56	3.01	
	MH-S E	2.82	3.69	3.25	
Figure 3C	J774 A	4.83	4.42	4.63	
	J774 B	0.68	1.53	1.10	
	J774 C	1.89	3.93	2.91	
	J774 D	2.36	4.72	3.54	
	J774 E	2.82	3.69	3.25	
Figure 4F	BCGlux - 48-500	1.10	3.19	2.14	
	BCGlux - 96-200	4.80	4.48	4.64	
	H37Rvlux - 48-500	2.42	8.21	5.32	
	H37Rvlux - 96-200	3.11	4.86	3.99	
Figure 6A	convMGIA	11.34	10.30	10.82	
	modMGIA	1.85	0.95	1.40	

Supplementary Table 2. Potential problems and proposed mitigations for troubleshooting

Potential problems	Mitigation/s
**No access to cell lines used in the study**	The target cell population could be enriched by using primary peritoneal mouse macrophages. To reduce use of animals, they could be recovered from the same animals that immune tissues are taken from. Though this adds an extra layer of work, the principle of the method (increasing sensitivity by enriching for target cells) is retained.<br><br>Other macrophage cell lines may also be used. However, initial optimisation experiments need to be carried out to determine the utility and permissiveness of the cell line to mycobacterial infection.
**Inconsistency of results**	This would most likely be caused by slight differences in experimental conditions. For example, physiological state of target macrophage cells may differ if recovered from exponential or stationary growth phase. Cell ratios may also impact readouts, as can the scale of culture. This can be mitigated against by optimising and accurately reproducing assay conditions.
**Lack of CL3 (containment level 3) facility**	We have demonstrated that BCG can also be used in both the conventional and modified assay, as a surrogate for Mtb. The readouts correlated well.
**No access to microbiological plating**	Additional readouts can be used, such as BACTEC MGIT assay (originally used in most conventional MGIA reports) but also luminescence-based readings, as demonstrated in this study.
**Difficult translation to human cells**	The modified assay cannot be easily translated to testing human immune cells. Whilst there are macrophage cell lines available (THP-1, U937), the genotypic (HLA) human diversity limits their application. A mitigation to this could be an enrichment of monocytic population from PBMC as the initial step, before setting up MGIA.
